# Antioxidant potential of *Sutherlandia frutescens* and its protective effects against oxidative stress in various cell cultures

**DOI:** 10.1186/1472-6882-14-271

**Published:** 2014-07-29

**Authors:** Shakila Tobwala, Weili Fan, Connor J Hines, William R Folk, Nuran Ercal

**Affiliations:** Department of Chemistry, Missouri University of Science and Technology, 400 West 11th Street, 142 Schrenk Hall, Rolla, MO 65409 USA; Department of Biochemistry, University of Missouri, Columbia, MO 65211 USA

**Keywords:** *Sutherlandia frutescens*, Oxidative stress, Antioxidant, Glutathione, Radical scavenging, Reactive oxygen species

## Abstract

**Background:**

*Sutherlandia frutescens* (L.) R.Br. (SF) is a South African plant that is widely used to treat stress, infections, cancer, and chronic diseases, many of which involve oxidative stress. The aim of the study was to quantitatively assess the antioxidant potential of SF extracts in cell-free system as well as in cell lines.

**Methods:**

Dried SF vegetative parts were extracted using six different solvents, and the extracts were assessed for total phenolic and flavonoid contents, total reducing power, iron chelating capacity, and free radical scavenging power, including, scavenging of hydroxyl radicals, superoxide anions, nitric oxide, and hydrogen peroxide. We further investigated the freeze-dried hot water extract of SF (SFE) to assess its effect against oxidative stress induced by *tert*-butyl hydroperoxide (t-BHP), an organic peroxide. Three different cell lines: Chinese hamster ovary (CHO), human hepatoma (HepaRG), and human pulmonary alveolar carcinoma (A549) cells, were employed to determine cell viability, intracellular reactive oxygen species (ROS) levels, and reduced to oxidized glutathione levels (GSH/GSSG).

**Results:**

The results indicated that: (1) SF extracts have significant antioxidant potential that is dependent upon the nature of the extraction solvent and (2) SFE protects against tBHP-induced oxidative stress in cells by scavenging ROS and preserving intracellular GSH/GSSG.

**Conclusion:**

Oxidative stress is implicated in a number of disorders, and due to the public’s concerns about synthetic antioxidants, various natural antioxidants are being explored for their therapeutic potential. Our findings support claims for *S. frutescens* being a promising adjunctive therapeutic for oxidative stress-related health problems.

## Background

Oxidative stress, the imbalance between antioxidant defense and oxidant production in cells, is implicated in the onset and progression of many health problems [1, 2]. One of the important effects of oxidative stress and free radical generation is decreased levels of cellular antioxidants. Changes in the redox state may affect signaling pathways for biologic processes and disrupt cellular functions.

A logical approach to treating oxidative stress-related disorders is the use of exogenous antioxidants. Many studies have been undertaken to evaluate the efficacy of synthetic and naturally occurring antioxidants in combating the damaging effects of free radicals and reactive oxygen species (ROS), and herbal antioxidants are of special interest to the public because of the perception of their lower toxicities compared to synthetic candidates [3]. The majority of natural antioxidants are polyphenols, which exhibit powerful antioxidant activity by acting as free radical scavengers, hydrogen donors, singlet oxygen quenchers, and metal ion chelators, in addition to inducing gene expressions of antioxidant enzymes [4–6].

*Sutherlandia frutescens* (SF) has been traditionally used in Africa in the treatment of a wide variety of stress related ailments, including cancer, diabetes, infections, and HIV/AIDS [7]. Phytochemical investigations of this plant showed that it contains significant amounts of gamma amino butyric acid and L-canavanine, pinitol, flavonol glycosides, and triterpenoid saponins [7], that may be pharmacologically relevant.

Flavonoids, the largest family of polyphenolic compounds, protect plants against parasites, oxidative injury, and harsh climatic conditions. They are divided into several subclasses: anthocyanins, flavanols, flavanones, flavonols, flavones, and isoflavones. Flavonoids exert their effects by neutralizing or chelating different types of radicals [8–10] and the position of hydroxyl groups and other features of the chemical structure are important for their antioxidant and free radical scavenging activities. Four 3-hydroxy-3-methylglutaroyl-containing flavonol glycosides, known as sutherlandins A – D have been isolated from SF [11].

The antioxidant potential of SF has been reported previously; however, it has not been extensively studied: Fernandes et al. reported that hot water extract of SF scavenges superoxide (O_2_^•^‾) and hydrogen peroxide (H_2_O_2_) in a cell-free system, as well as in presence of human neutrophils [12]: in addition, Katerere and Eloff investigated the antibacterial and antioxidant activity of SF [13], and Koleva et al. reported substantial radical scavenging activity by SF extracts [14].

The therapeutic claims made about SF for a wide variety of ailments inspired us to evaluate its antioxidant potential. In the present study, dried vegetative parts of SF were extracted by methanol, ethanol, acetone, acetonitrile, hot water, and cold water homogenization. To quantitatively assess the antioxidant potential of SF extracts, we used several tests in cell-free systems as well as in cell cultures. The extracts were examined for different ROS scavenging activities, including hydroxyl, superoxide, nitric oxide, and hydrogen peroxide, in addition to total phenolic and flavonoid content, iron chelating capacity, and reducing power. We further investigated the freeze-dried hot water extracts of SF (SFE) to assess effects on cell viability, intracellular ROS levels, and GSH/GSSG ratios of Chinese hamster ovary (CHO), human hepatoma (HepaRG), and human pulmonary alveolar carcinoma (A549) cells. These findings support claims for SF having the potential as an herbal antioxidant.

## Methods

### Chemicals

All chemicals used for analytical purposes were obtained from Sigma (St. Louis, MO) and Fisher Scientific (Fair Lawn, NJ). The human hepatoma cells (HepaRG) were obtained from Invitrogen. Chinese hamster ovary (CHO) K1 and the human lung carcinoma pulmonary type II-like epithelium cells were obtained from American Type Culture Collection (ATCC) (Manassas, VA, USA).

### Preparation of plant extracts

Dried, milled vegetative parts of *Sutherlandia frutescens* (family: Fabaceae/Leguminosa), obtained from Big Tree Nutraceutical, Fish Hoek, South Africa were extracted in six different solvents: methanol, ethanol, acetone, acetonitrile, hot water, and cold water homogenization. Briefly, 1 g of dried SF was extracted in 50 ml of each solvent. Extraction in methanol, ethanol, acetone, and acetonitrile was done by adding respective solvent to the SF, followed by sonication for 20 min. Hot water extract was prepared by boiling SF for 20 min and cooling to room temperature. Cold water extract was prepared by homogenizing SF in water by tissue tearor (Biospec Products) for 20 min. All extracts were vacuum filtered and thereafter stored at 4°C until further use (20 mg/ml). For studies in cells, however, the hot water filtrate was freeze-dried for 72 h in the Savant refrigerated vapor trap (RVT4104-180) to obtain a dried powdered plant extract. Lyophilized extract was then dissolved in a serum-free media to a final concentration of 1 mg/ml stock solution, referred as a SFE (a yield of 5%).

### Determination of total polyphenolic content

Total phenolic content of the SF extract was determined, as described by Konaté et al. with minor modifications [15], which rely on the formation of a bluish-grey complex between Folin-Ciocalteu reagent (F-C reagent) with phenols. Briefly, 125 μl of the plant extract was mixed with 625 μl of F-C reagent (10-fold diluted) and incubated at room temperature for 5 min, followed by the addition of 500 μl of 75 mg/ml Na_2_CO_3_ solution. The mixture was vortexed and incubated at room temperature in the dark for 90 min. The absorbance at 760 nm was measured against a reagent blank, with gallic acid used as the standard. The results were expressed as μg of gallic acid equivalents (GAE)/mg of dried plant material.

### Determination of total flavonoid content

Determination of the total flavonoid content of the extracts, which was as described by Kalava et al. with minor modifications, relied on the formation of an acid-stable, bright yellow complex between aluminum chloride and flavones/flavonoids [16]. In brief, 200 μl of plant extract was mixed with a solution of 60 μl of 5% NaNO_2_ and 800 μl of deionized water, and vortexed, then allowed to stand at room temperature for 5 min in the dark. Thereafter, 60 μl of 10% AlCl_3_ were added to the mixture, followed by 5 min of incubation at room temperature in the dark. The color was developed by adding 400 μl of 1 M NaOH and A _415 nm_ was measured against a reagent blank with quercetin as a standard. The concentrations of phenols in the test samples were calculated from the calibration plot and expressed as μg of quercetin equivalents (QE)/mg of dried plant leaves.

### Determination of total reducing power

Total reducing power of the SF extracts was determined according to the method of Jayanthi et al. [17]; a solution comprising 2.5 ml of 0.2 M phosphate buffer (pH 6.6) and 2.5 ml of 1% potassium ferricyanide were added to 1 ml plant extract and gently mixed. The mixtures were incubated at 50 ^0^C in a water bath for 20 min, then mixed with 2.5 ml of 10% trichloroacetic acid (TCA) and centrifuged at 6,000 rpm for 10 min. From the top layer, 2.5 ml were transferred into tubes containing 2.5 ml distilled water and 0.5 ml of 0.1% ferric chloride (FeCl_3_.6H_2_O). The resulting solutions were mixed well and, after 5 min, the absorbance was measured at 700 nm with ascorbic acid as the standard. Results were expressed as milligrams of ascorbic acid equivalent (AAE)/mg of dried plant material.

### Determination of radical scavenging power

The radical scavenging power of extracts was assessed by the method of Shyamala et al. [18] with slight modifications. The reaction mixture had a total volume of 3 ml, which included 2.9 ml of DPPH (1 × 10^-4^ M DPPH) and 0.1 ml of the corresponding sample at various concentrations. The solutions were left in the dark, at room temperature for 30 min, and the resulting absorbance at 520 nm was measured against blanks. Decrease in intensity corresponded to a higher radical scavenging power, calculated as [1 - *A*1÷*A*2] × 100%, whereas A1 and A2 are the absorbance with and without plant extract, respectively. Butylated hydroxytoluene (BHT) was used as the standard.

### Determination of H_2_O_2_ scavenging power

The H_2_O_2_ scavenging power of the extracts was determined as described by Ozyurek et al. with modifications [19], based on the complex between neocupronine, a cuprous ion-specific chromogen, and cuprous ion, the reduction product of cupric ion by hydrogen peroxide. In brief, 500 μl phosphate buffer (200 mM; pH = 7.4), 400 μl of 10 mM H_2_O_2_ or deionized water, 200 μl of extract or solvent, and 400 μl of 0.1 mM CuCl_2_ were mixed and incubated at 37°C for 30 min. Then, 400 μl of deionized water were added and 500 μl of this solution was added to a mixture of 1 ml of 10 mM CuCl_2_, 1 ml of 7.5 mM neocupronine, and 2 ml of 1 M NH_4_Ac. Absorbance was measured at 450 nm and the hydrogen peroxide scavenging power was calculated as [1 - (*A*1 ÷ *A*2)/*A*0] × 100%, where A0 was the absorbance of the mixture without extract but with H_2_O_2_, A1 was that with both the extract and H_2_O_2_ and A2 was that with extract but without H_2_O_2_. Pyruvate was used as the standard.

### Determination of nitric oxide scavenging power

Nitric oxide scavenging power of the plant extract was determined as described by Kumar et al. [20] with slight modifications, was based on the formation of a diazo compound between Griess Reagent and nitrate, the oxidation product of nitric oxide released by sodium nitroprusside. Briefly, 1 ml of the extract or solvent was mixed with 0.3 ml of 60 mM sodium nitroprusside, and illuminated under fluorescent light at room temperature for 150 min. Thereafter, 0.5 ml of Griess Reagent [1% sulfanilamide, 2% phosphoric acid and 0.1% N-(1-naphthyl)-ethylenediamine∙2HCl] or deionized water was added. Absorbance was measured at 546 nm and nitric oxide scavenging power was calculated as [1 - (*A*1÷*A*2)/*A*0] × 100% where A0 is the absorbance of the mixture without extract, A1 is the absorbance with both, and A2 is the absorbance with extract and without Griess Reagent. Curcumin was used as the standard.

### Determination of O_2_^•^‾ scavenging power

The O_2_^•^‾ scavenging power of the extract, determined as described by Bajpai et al. [21] with slight modifications, was based on reduction of nitroblue tetrazolium (NBT) to a purple product by superoxide anion, which was generated via a redox cycle by 5-methyl-phenazinium methyl sulfate (PMS). In brief, 1 ml of 156 μM NBT (in 100 mM phosphate buffer, pH 7.4) or buffer alone, 1 ml of 486 μM NADH (in buffer) or buffer, and 100 μl of extract or solvent were mixed. 100 μl of 330 μM PMS (in buffer) or buffer alone were added and the mixture was incubated at room temperature for 5 min. Absorbance was measured at 560 nm and the superoxide scavenging power was calculated as [1 - (*A*1÷*A*2)/*A*0] × 100%, where A0 is the absorbance of the mixture without extract but with NBT/NADH, A1 is the absorbance with extract and NBT/NADH and A2 is the absorbance with extract and without NBT/NADH. Quercetin was used as the standard.

### Determination of ^•^OH scavenging power

The ^•^OH scavenging power of the extracts was determined as described by Kunchandy et al. [22] with slight modification, was based on the formation of a complex between thio barbituric acid (TBA) and the oxidation product of 2-deoxyribose by hydroxyl radicals. In brief, 100 μl of 28 mM 2-deoxyribose or deionized water, as well as 500 μl of 20 mM phosphate buffer (pH 7.4) were added to 100 μl extract or solvent. Thereafter, 100 μl of 1mM FeSO_4_, 100 μl of 1 mM EDTA tetrasodium salt, and 100 μl of 10 mM H_2_O_2_ were added to the mixture, which was incubated at 37°C for 1 h. Then, 2 ml of 2.8% TCA and 2 ml of 1% TBA were added. This mixture was boiled for 15 min and allowed to cool to room temperature and A_532nm_ measured. Hydroxyl radical scavenging power of the extract was calculated as [1 - (*A*1÷*A*2)/*A*0] × 100% where A0 is the absorbance of the mixture without extract but with deoxyribose, A1 is the absorbance with both, and A2 is the absorbance without deoxyribose but with extract. Mannitol was used as the standard.

### Determination of iron (Fe^2+^) - chelating power

The iron-chelating power of the extracts, based on the reaction between ferrous ion and ferrozine was determined as described by Ercal et al. [23] with minor modifications. In brief, 100 μl of the extract (or solvent) were mixed with 100 μl of 0.6 mM FeSO_4_ and 1.7 ml of deionized water and incubated at room temperature for 5 min in the dark. Afterwards, 100 μl of a 5 mM ferrozine solution (in methanol or methanol) were added to the mixture and incubated for 5 min in the dark. Absorbance at 562 nm was measured and the chelating power of the plant extract was calculated as [1 - (*A*1÷*A*2)/*A*0] × 100%, where A0 is the absorbance of the control (without extract), A1 is the absorbance in the presence of the extract and A2 is the absorbance without ferrozine. EDTA tetrasodium salt was used as the standard.

### Cell culture

The human lung carcinoma pulmonary type II-like epithelium cells (A549) were seeded in 25 cm^2^ flasks coated with type 1 rat tail collagen (Sigma-Aldrich, St. Louis, MO) and maintained in F-12 Ham’s medium with 10% heat-inactivated fetal bovine serum in humidified 5% CO_2_/95% air at 37°C. The culture medium was changed every 3 days.

Human hepatoma cells (HepaRG) were seeded in 75 cm^2^ flasks coated with type 1 rat tail collagen (Sigma-Aldrich, St. Louis, MO) and maintained in William’s E medium supplemented with 10% FCS, 100 U penicillin, 100 μg/ml streptomycin, 5 ug/ml insulin, and hydrocortisone in humidified 5% CO2/95% air at 37°C. The culture medium was renewed every 3 days. After about 2 weeks, when the cells were confluent they were shifted to the same medium supplemented with 2% DMSO (differentiation medium). The medium was renewed every 2 to 3 days for 2 more weeks, then switched to a DMSO-free medium for 1 day prior to the cells being used for assays.

Chinese hamster ovary (CHO) K1 cells were grown in Ham’s F-12 culture medium, supplemented with 10% (v/v) fetal bovine serum (FBS), and 2 mM L-glutamine, 100 U/ml penicillin and 100 μg/ml streptomycin. The cells were maintained in a humidified incubator at 37°C and supplied with 95% O_2_ and 5% CO_2_.

### Determination of cell viability

Cells were seeded in 96-well plates at a density of approximately 1.25 × 10^4^ cells/well, for a day. To assess cytotoxicity of the SF extract, the cells were incubated with concentrations of SFE, ranging from 10 μg/ml to 1 mg/ml, in serum-free medium for 24 h. To assess cytotoxicity of t-BHP, the media were replaced by various concentrations of t-BHP (10 μM to 500 μM) in serum-free medium for 24 h. The protective effects of SFE were assessed by pretreating cells with 500 μg/ml of SFE for 2 h, followed by treatment with t-BHP for 24 h. After 24 h of t-BHP treatment, the medium was discarded and viability assessed with a Calcein AM assay KIT (Biotium, Inc. CA). The cells were washed three times with PBS, and 100 μl of 2.0 μM Calcein AM in PBS were added to each well for 30 min at 37°C. The fluorescence was measured with an excitation wavelength at 485 nm and an emission wavelength of 530 nm, using a microplate reader (FLUOstar, BMG Labtechnologies, Durham, NC, USA).

### Measurement of intracellular ROS levels

Intracellular ROS generation was measured using a well-characterized probe, 2′, 7′-dichlorodihydrofluorescein diacetate (H_2_DCF-DA) (Wang and Joseph, 1999). In brief, H_2_DCF-DA is diffused into cells and is deacetylated by cellular esterases to non-fluorescent 2′, 7′-dichlorodihydrofluorescein, which is rapidly oxidized to highly fluorescent 2′, 7′-dichlorofluorescein (DCF) by ROS. The fluorescence intensity is proportional to the ROS levels within the cell cytosol. In the groups with SFE pretreatment, media containing various concentrations of SFE was added and incubated for 2 h. Once pretreated, the cells were washed twice with PBS and incubated with a solution of 50 μM H_2_DCF-DA in phenol red free media for 30 min. This was followed by washing the cells twice with PBS. The respective groups were then dosed either with t-BHP or plain media for 24 h and fluorescence was determined at 485 nm excitation and 520 nm emission, using a microplate reader (FLUOstar, BMG Labtechnologies, Durham, NC, USA).

### Determination of intracellular glutathione (GSH) levels

Intracellular GSH content was determined by reverse phase HPLC. The protective effects of SFE were studied by pretreating cells with 500 μg/ml of SFE for 2 h, followed by treatment with t-BHP for 24 h. All cell samples were homogenized in SBB. Twenty microliters of this homogenate were added to 230 μl of HPLC grade water and 750 μl of NPM (1 mM in acetonitrile). The resulting solutions were incubated at room temperature for 5 min. The reaction was stopped by adding 10 μl of 2 N HCl. The samples were filtered through a 0.45 μm filter (Advantec MFS, Inc. Dulin, CA, USA) and injected onto the HPLC system. 5 μl of the sample were injected for analysis using a Thermo Finnigan TM Spectra SYSTEM SCM1000 Vacuum Membrane Degasser, Finnigan TM SpectraSYSTEM P2000 Gradient Pump, Finnigan TM SpectraSYSTEM AS3000 Autosampler, and FinniganTM SpectraSYSTEM FL3000 Fluorescence Detector (λex=330 nm and λem=376 nm). The HPLC column was a Reliasil ODS-1 C18 column (Column Engineering, Ontario, CA, USA). The mobile phase was 70% acetonitrile and 30% water and was adjusted to a pH of 2.5 through the addition of 1 ml/L of both acetic and o-phosphoric acids. The NPM derivatives were eluted from the column isocratically at a flow rate of 1 ml/min.

### Determination of glutathione disulfide (GSSG) levels

Total glutathione content was determined by reverse phase HPLC. Cell samples were homogenized in SBB. Twenty microliters of this homogenate were added to 60 μl of NADPH (2 mg/ml) in nanopure water and 20 μl of 1 unit/ml glutathione reductase were added to reduce GSSG. After 10 min of incubation at room temperature, the treated samples were diluted with 150 μl H_2_O, and then immediately derivatized with 750 μl of 1.0 mM NPM. The samples were analyzed by reverse phase HPLC as detailed for the determination of GSH. Data from the original and total current GSH levels in each sample were subsequently used to calculate the levels of GSSG present in each sample.

### Determination of protein

Protein levels of the cell samples were measured using the Bradford method [24]. Concentrated Coomassie blue (Bio-Rad,Hercules, CA, USA) was diluted 1:5 (v/v) with distilled water and 1.5 ml added to 50 μl of diluted cell homogenate; the solution was vortexed and allowed to stand at room temperature for 10 min and the absorbance was measured at 595 nm using bovine serum albumin as the protein standard.

### Statistical analysis

All reported values were represented as the mean ± S.D (n=4). Statistical analyses was performed using GraphPad Prism software (GraphPad, San Diego, CA). Statistical significance was ascertained by one way analysis of variance, followed by Tukey’s multiple comparison tests. Values of p<0.05 were considered significant.

## Results

### Total phenolic content

Total phenolic content, expressed as μg gallic acid equivalent/mg dried SF, was determined by the polarity of the extraction solvent with the following order (from high to low): Hot water > cold water > methanol >ethanol > acetone > acetonitrile (Table 1). Hot water appears to be the best extraction solvent for polyphenols with the total phenolic content of hot water extract being 12.9 ± 0.17μg gallic acid equivalent/mg of dried SF.

**Table 1:** **Total polyphenolic content, flavonoid contents, and reducing power of various extracts of** ***Sutherlandia Frutescens***

Extract	Total Polyphenolic Content (GAE, in μg/mg of dried leaves)	Flavonoid Content (QE, in μg/mg of dried leaves)	Total Reducing Power (AAE, in μg/mg of dried leaves)
Hot water	12.9 ± 0.17	28.7 ± 0.32	8.63 ± 0.17
Cold water	11.3 ± 0.32^a^	17.5 ± 0.50^a^	7.74 ± 0.09^a^
Methanol	9.26 ± 0.18^ab^	24.7 ± 0.32^ab^	7.10 ± 0.47^ab^
Ethanol	4.66 ± 0.13^abc^	17.0 ± 0.41^ac^	2.06 ± 0.09^abc^
Acetone	2.29 ± 0.16^abcd^	9.33 ± 0.38^abcd^	0.98 ± 0.16^abcd^
Acetonitrile	1.57 ± 0.01^abcde^	6.08 ± 0.18^abcde^	0.68 ± 0.05^abcd^

All experiments were performed in quadruplicates, and the values reported are mean ± SD. (a: different from hot water extract, b: different from cold water extract, c: different from methanol extract, d: different from ethanol extract, e: different from acetone extract, p<0.05).

### Total flavonoid content

Similar to the phenolic content, the total flavonoid content was affected by the polarity of the extraction solvent with the following order: hot water > methanol > cold water/homogenization ≥ ethanol > acetone > acetonitrile (Table 1). The total flavonoid content of the hot water extract of SF was 28.7 ± 0.324 μg quercetin equivalent/mg of dried SF.

### Reducing power

Reducing power is associated with antioxidant activity and may serve as an important index of the antioxidant potential. Reducing power characteristics of different solvent extracts of SF are summarized in Table 1 in the following order: hot water > cold water > methanol > ethanol > acetone > acetonitrile. Total reducing power of hot water extract was 8.63 μg ascorbic acid equivalent/mg of dried SF, only 1.11 times higher than that of cold water-homogenization and 1.21 times higher than methanol extract. In contrast, the values of ethanol, acetone, and acetonitrile were 4.19 times, 8.80 times and 12.74 times lower than that of hot water extract, respectively.

### Radical scavenging power

**Figure 1:**
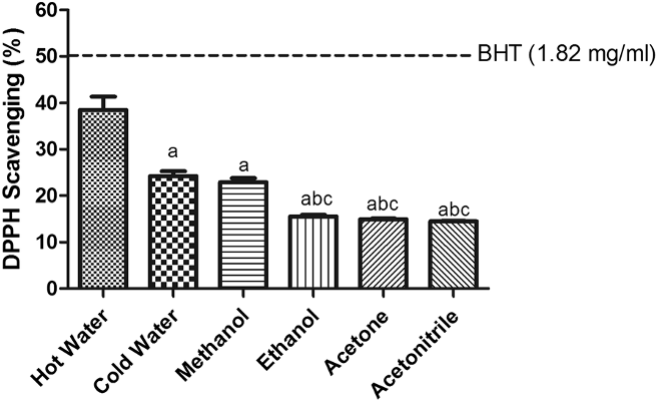
**The DPPH radical-scavenging activity of SF extracts.** The absorbance values were converted to the scavenging effect (%) and data plotted as the means of the replicate scavenging effect (%) values ± S.D. (n = 4). The IC_50_ value of the reference compound BHT was 1.82 mg/ml. The concentration of SF extract was 20 mg of dried leaves/ml. (a: different from hot water extract, b: different from cold water extract, c: different from methanol extract, d: different from ethanol extract, e: different from acetone extract, p<0.05).

Free radical scavenging power is an important property for consideration in evaluating the protective effects of an antioxidant because of the deleterious effects of radicals in biological systems. The DPPH scavenging method is a simple method for assessing the radical scavenging power of a compound [25], and is based on the ability of antioxidants to reduce the DPPH radical to a more stable DPPHH form. In the presence of a free radical scavenger, DPPH is reduced with a corresponding decrease in absorbance. BHT, a derivative of phenol and a functionally synthetic analogue of Vitamin E that suppresses autoxidation, was used as the standard. The DPPH scavenging activities of different SF extracts (Figure 1) are affected by the extraction solvent as follows: hot water > cold water > methanol > ethanol ≥ acetone ≥ acetonitrile. Hot water extract possessed the highest radical scavenging ability (39%) among all SF extracts.

### H_2_O_2_ scavenging capacity

**Figure 2:**
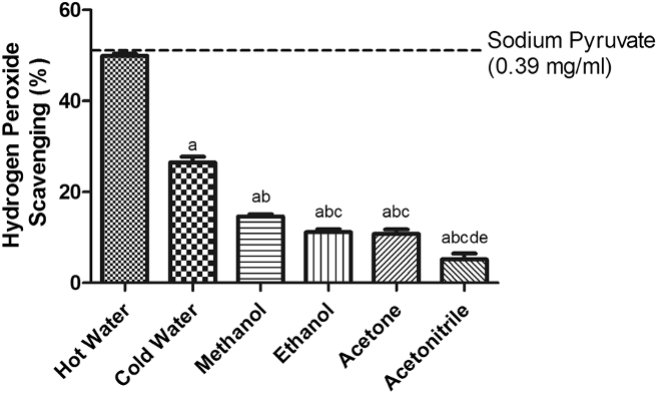
**The hydrogen peroxide radical-scavenging activity of SF extracts.** The absorbance values were converted to the scavenging effect (%) and data plotted as the means of the replicate scavenging effect (%) values ± S.D. (n = 4). The IC_50_ value of the reference compound sodium pyruvate was 0.39 mg/ml. The concentration of SF extract was 20 mg of dried leaves/ml. (a: different from hot water extract, b: different from cold water extract, c: different from methanol extract, d: different from ethanol extract, e: different from acetone extract, p<0.05).

H_2_O_2_ is not a direct reactive oxygen species but, due to its high membrane permeability, it enters cells and leads to the production of hydroxyl radicals and superoxide radicals in the presence of metal ions. Thus, an important measure of the antioxidant activity of a compound is its scavenging activity for H_2_O_2_. As compared with sodium pyruvate, the H_2_O_2_ scavenging power of extracts was: hot water > cold water > methanol > ethanol ≥ acetone > acetonitrile (Figure 2).

### ^•^OH scavenging capacity

**Figure 3:**
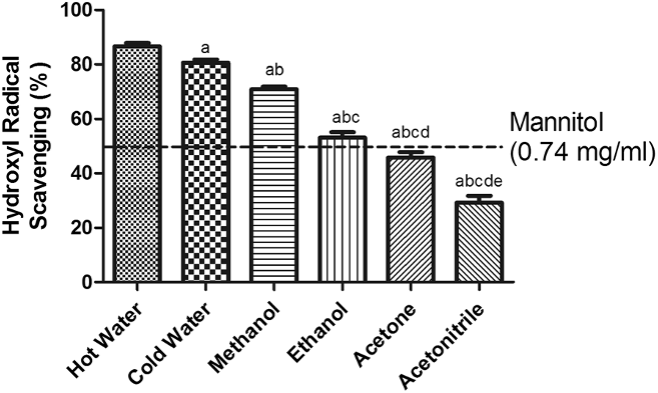
**The hydroxyl radical-scavenging activity of SF extracts.** The absorbance values were converted to the scavenging effect (%) and data plotted as the means of the replicate scavenging effect (%) values ± S.D. (n = 4). The IC_50_ value of the reference compound mannitol was 0.74 mg/ml. The concentration of SF extract was 20 mg of dried leaves/ml. (a: different from hot water extract, b: different from cold water extract, c: different from methanol extract, d: different from ethanol extract, e: different from acetone extract, p<0.05).

^•^OH radicals are short lived and can be highly deleterious to cell membranes and other biomolecules. ^•^OH radical scavenging is therefore necessary to protect cells from oxidative damage. Of the many different ways by which ^•^OH radicals can be produced, the most important is the Fenton reaction, which involves the transition metal catalyzed decomposition of hydrogen peroxide to produce hydroxyl radicals [26]. Mannitol was used as a standard antioxidant for comparison, with the hydroxyl radical scavenging capacity of extracts following the order: hot water > cold water > methanol > ethanol > acetone > acetonitrile (Figure 3).

### Superoxide radical anion scavenging capacity

**Figure 4:**
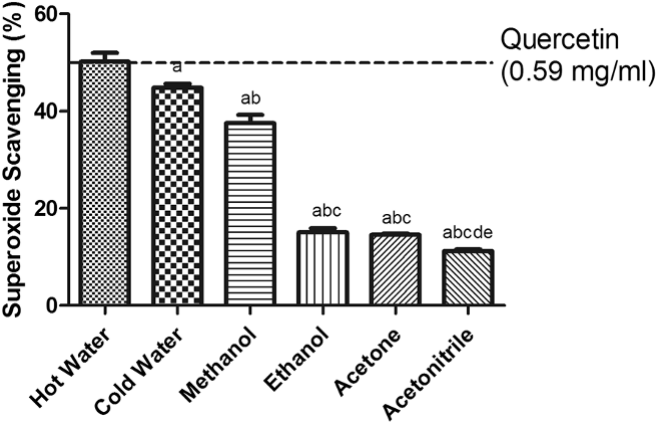
**The superoxide-scavenging activity of SF extracts.** The absorbance values were converted to the scavenging effect (%) and data plotted as the means of the replicate scavenging effect (%) values ± S.D. (n = 4). The IC_50_ value of the reference compound quercetin was 0.59 mg/ml. The concentration of SF extract was 20 mg of dried leaves/ml. (a: different from hot water extract, b: different from cold water extract, c: different from methanol extract, d: different from ethanol extract, e: different from acetone extract, p<0.05).

Superoxide radical anion (O^•^‾) originates from the one-electron reduction of free molecular oxygen, and is implicated in a number of oxidative stress related disorders. The superoxide scavenging activities of the samples (Figure 4) were in the following order (from highest to lowest): hot water > cold water/homogenization >methanol > ethanol ≥acetone > acetonitrile.

### Nitric oxide (NO) scavenging capacity

**Figure 5:**
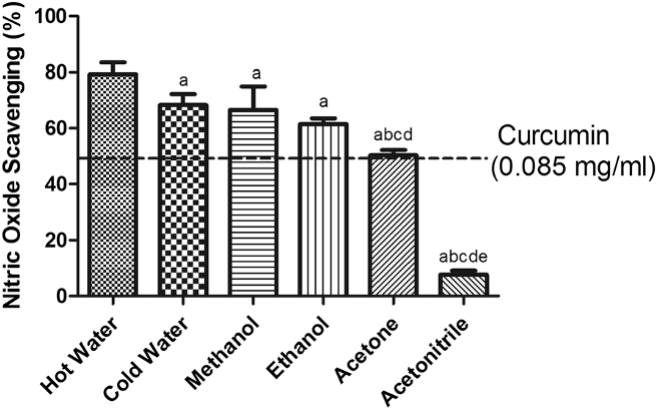
**The nitric oxide-scavenging activity of SF extracts.** The absorbance values were converted to the scavenging effect (%) and data plotted as the means of the replicate scavenging effect (%) values ± S.D. (n = 4). The IC_50_ value of the reference compound curcumin was 0.085 mg/ml. The concentration of SF extract was 20 mg of dried leaves/ml. (a: different from hot water extract, b: different from cold water extract, c: different from methanol extract, d: different from ethanol extract, e: different from acetone extract, p<0.05).

NO is a short-lived (half-life 3–30 s) lipophillic colorless gas that can very easily diffuse between cells. Although it does not interact directly with biomolecules, NO can react with oxygen to produce stable intermediates, such as NO_2_, N_2_O_4_, N_3_O_4_[27], and peroxynitrite upon reaction with superoxide [28], which are in turn deleterious. The NO scavenging ability of the extracts followed this order (from high to low): hot water > cold water/homogenization ≥ methanol ≥ ethanol > acetone > acetonitrile (Figure 5).

### Iron (Fe^2+^) - chelating capacity

**Figure 6:**
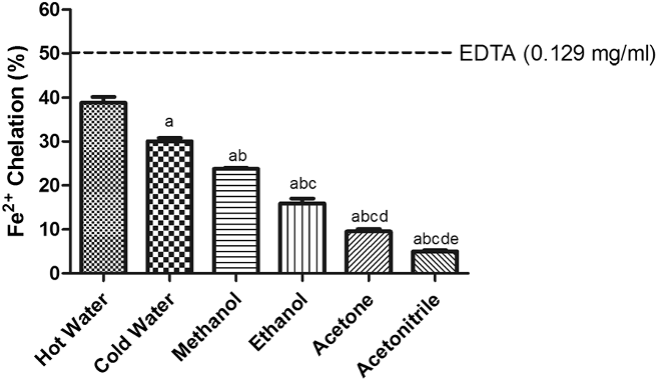
**Fe** ^**2+**^ **-chelating activity of SF extracts.** The absorbance values were converted to the scavenging effect (%) and data plotted as the means of the replicate chelating effect (%) values ± S.D. (n = 4). The IC_50_ value of the reference compound EDTA was 0.129 mg/ml. The concentration of SF extract was 20 mg of dried leaves/ml. (a: different from hot water extract, b: different from cold water extract, c: different from methanol extract, d: different from ethanol extract, e: different from acetone extract, p<0.05).

Metal chelating property is especially important because of the ability of transition metal ions like Fe^2+^ to catalyze a number of free radical generating reactions such as the Fenton reaction. ^•^OH produced as a result of this reaction can accelerate lipid peroxidation by decomposing lipid hydroperoxides into peroxyl and alkoxyl radicals that can abstract hydrogen, propagate the chain reaction [29], and damage cell membranes. Metal chelating activity is, therefore, an important indicator of the antioxidant capacity of a compound. In the presence of a chelating agent, Fe^2+^ is no longer available to form a colored complex, as reflected by a decrease in absorbance. The iron chelating ability was in the following order: hot water > cold water/homogenization >methanol > ethanol >acetone > acetonitrile (Figure 6).

### Cytotoxicity of SFE on A549, HepaRG, and CHO cells

**Figure 7:**
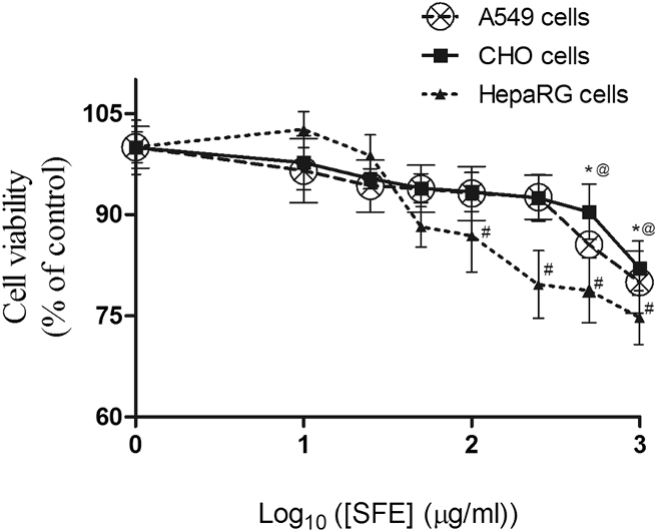
**Dose response curves to compare the effects of SFE on cell viability in A549, CHO, and HepaRG cell lines.** These cell lines were treated with various doses of SFE for 24 h and viability was determined by Calcein AM assay. Values represent mean ± SD (n=5).

Since hot water extract appeared to have high antioxidant potential, it was chosen for subsequent cell based studies. SFE was not toxic to CHO cells or A549 cells up to a concentration of 500 μg/ml for 24 h, however, a decrease in cell viability was observed for HepaRG cells above 100 μg/ml of SFE treatment for 24 h (Figure 7). This was confirmed using a calcein AM assay.

### Effect of SFE on t-BHP-induced cytotoxicity

**Figure 8:**
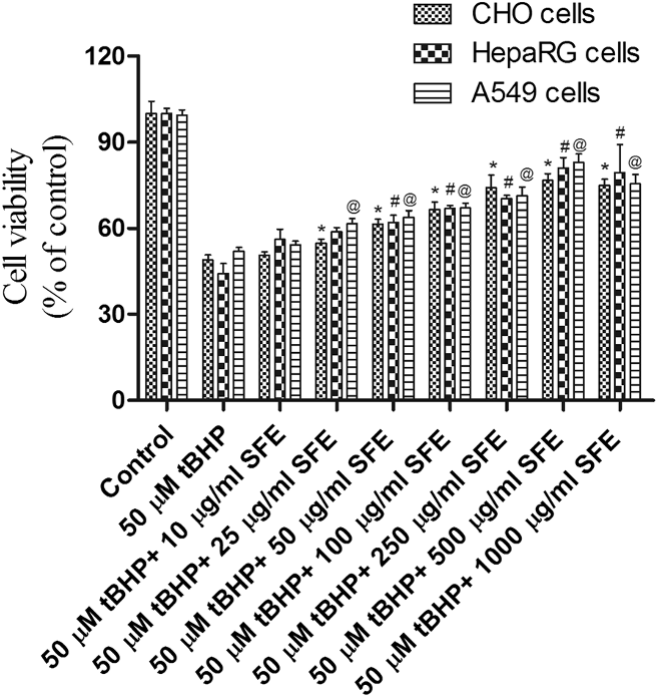
**Protection against tBHP-induced oxidative stress by SFE.** Cell viability was quantified by a Calcein AM assay 24 h after exposure to t-BHP, following a 2 h pretreatment with SFE. Treatment with t-BHP (50 μM) alone was seen to significantly decrease cell viability. SFE showed protection against t-BHP-induced cell toxicity in a dose-dependent manner. Values represent mean ± SD (n=5).

To study the protective effects of SFE on t-BHP-induced toxicity, cells were pretreated with various concentrations of SFE (10 μg/ml to 1000 μg/ml) for 2 h, followed by incubation with 50 μM of t-BHP for 24 h. In all three cell lines, cell viability decreased to approximately 40-50% of the control, when treated with 50 μM t-BHP, which increased significantly in a dose dependent manner to 500 μg/ml upon pretreatment with SFE (Figure 8). However, no further increase was observed upon increasing the concentration to 1000 μg/ml. The lowest nontoxic concentration of SFE that provided maximum protection was 500 μg/ml and, therefore, it was chosen for subsequent experiments to study the protective effect of SFE on tBHP-induced GSH depletion.

### Effect of SFE on intracellular ROS levels

**Figure 9:**
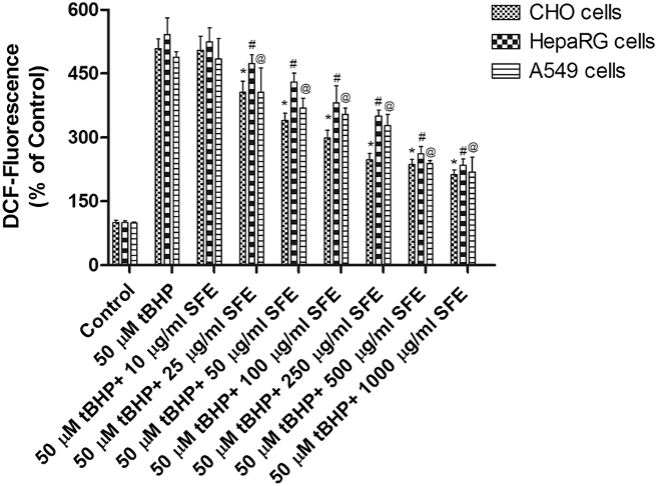
**Intracellular ROS levels. ROS levels after pretreatment with various concentrations of SFE for 2 h followed by treatment with t-BHP (50 μM) for 2h.** Treatment with 50 μM t-BHP significantly increased the ROS levels. Pretreatment with SFE decreased the ROS levels in a dose-dependent manner. Values represent mean ± SD (n=5).

To test the hypothesis that SFE combat oxidative stress by scavenging ROS, we measured ROS levels after the exposure of cells to 50 μM t-BHP for 2 h. A dose dependent increase in the production of ROS was observed in all three cell lines with exposure to tBHP (data not shown). To study the protective effects of SFE on a tBHP- induced increase in ROS levels, all three cell lines were pretreated with various concentrations of SFE for 2 h, followed by incubation with 50 μM of tBHP for 2 h. There was a significant dose dependent decrease in levels of ROS with an increase in the concentration of SFE (Figure 9).

### Effect of SFE on intracellular glutathione levels

**Figure 10:**
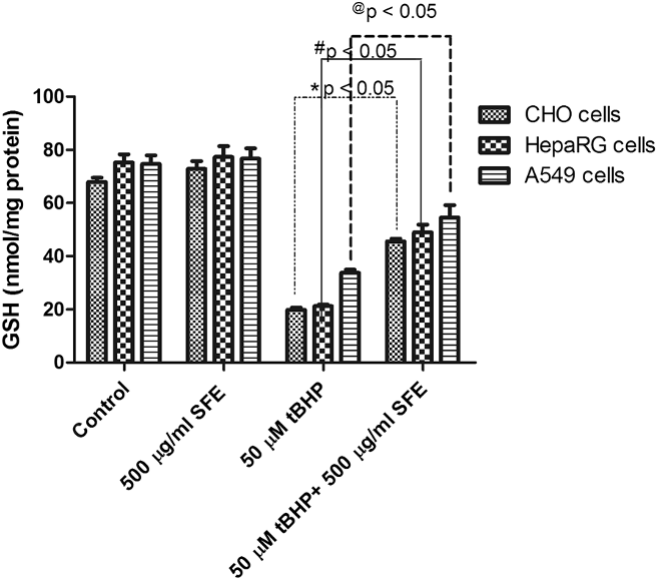
**Intracellular GSH levels in A549, HepaRG, and CHO cells after treatment with 50 μM t-BHP and 500 μg/ml SFE.** GSH levels were measured after 24 h of treatment for control, SFE, t-BHP, and t-BHP + SFE groups. Exposure to t-BHP (50 μM) significantly decreased intracellular GSH level. Pretreatment with SFE (500 μg/ml) 2 h before the addition of t-BHP, prevented such a dramatic decrease. *p ≤ 0.05 compared to t-BHP group.

To examine whether SFE acts as an antioxidant by scavenging ROS, thereby preventing further GSH depletion, we measured the levels of intracellular GSH. Figure 10 shows the effect of tBHP on intracellular GSH levels in the presence and absence of a SFE. A 24 h exposure with 50 μM of tBHP decreased the GSH level by more than 50% of that of the control in all the three cell lines studied. Pretreatment with 500 μg/ml of SFE increased the GSH level significantly in all three cell lines (Figure 10).

### Effect of SFE on glutathione disulfide levels and GSH/GSSG ratio

**Figure 11:**
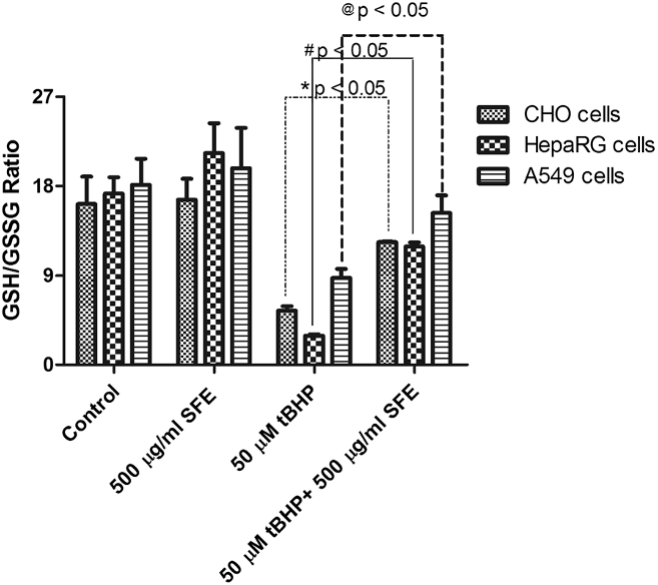
**Intracellular GSH/GSSG ratios in A549, HepaRG, and CHO cells after treatment with 50 μM t-BHP and 500 μg/ml SFE.** GSH/GSSG ratios were determined after 24 h of treatment for control, SFE, t-BHP, and t-BHP + SFE groups. Exposure to t-BHP (50 μM) significantly decreased intracellular GSH/GSSG ratio. Pretreatment with SFE (500 μg/ml) 2 h before the addition of t-BHP, prevented such a dramatic decrease. *p ≤ 0.05 compared to t-BHP group.

The GSH/GSSG ratio decreased significantly upon treatment with 50 μM t-BHP in all three cell lines. However, pretreatment with 500 μg/ml of SFE significantly decreased the level of GSSG and increased the ratio of GSH/GSSG to approximately that of the control group (Figure 11).

## Discussion

Aspects of the antioxidant role of SF have been studied previously [12–14]; however, an extensive study comparing the antioxidant potential of different solvent extracts of SF in a cell-free system and in cell lines has not been reported. Here, we report the antioxidant potential of SF extracts as well as their protective role in t-BHP-induced oxidative stress in three cell lines.

The extracting solvent significantly affected the total phenolic content, flavonoid content, reducing power, and the radical scavenging activity of SF extracts. The yield of SF hot water extract was about 5% (weight of lyophilized extract/weight of dried plant material). Polarity of the solvent affected the antioxidant potential of the extracts with hot water being the best solvent for extracting total phenolics, including flavonoids. Our results are similar to those of Katerere and Eloff, who investigated the antibacterial and antioxidant activity of SF using two different extraction schemes. They reported substantial radical scavenging activity in the more polar extracts, attributed to the polar phenolic compounds [13]. Contrary to this, Koleva et al. reported the highest radical scavenging activity in the methanol, ethyl acetate, and 1-butanol extracts and lowest in the aqueous extract, which was attributed to the origin of the plant sample and not the solvent polarity. Their semi-quantitative TLC tests showed smaller amounts of phenolic components in these more polar extracts [14].

Our results indicate that SF plant extract contains significant amounts of flavonoids, whose mechanism of action is through scavenging or chelation [30]. It appears that hot water extract has the highest radical scavenging power, and is more powerful than the BHT standard, considering that the actual yield of hot water extract is 5% (20 mg of dried plant material yield 1 mg of the lyophilized hot water extract). However, it does not show good reducing power and it appears that the superior radical scavenging capacity of hot water extract might provide it with significant antioxidant properties.

Superoxide anion, together with its dismutation product, hydrogen peroxide, is deleterious to macromolecules [31]. Flavonoids are effective scavengers of superoxide anions and thereby protective against oxidative damage [8]. Our results suggest that SF extract is a potent scavenger of superoxide radicals and hydroxyl radicals. Hydroxyl radicals are one of the major reactive oxygen species produced as a result of Fenton’s reaction, causing lipid peroxidation and subsequent cellular damage [26]. Hot water plant extract proved to be the most potent scavenger of hydroxyl radicals, followed by superoxide anion radical and then hydrogen peroxide. However, it did not exhibit considerable nitric oxide scavenging ability. Our results concur with Fernandes et al., who reported that extracts from hot water demonstrated superoxide and hydrogen peroxide scavenging activity, and attributed the antioxidant activity of SF hot water extract to the phenolic compounds [12].

Iron can stimulate lipid peroxidation via Fenton reaction and also by decomposing lipid hydroperoxides into peroxyl and alkoxyl radicals that can further propagate the chain reaction [29]. According to our results, the metal chelating activity of SF extract is not as good as the standard EDTA and, therefore, its antioxidant potential could be attributed primarily to its radical scavenging power. Differences in the antioxidant potential of extracts of SF could be attributed to the differences in the composition of the extracts in those solvents.

SFE were not toxic to CHO and A549 cells below a concentration of 500 μg/ml. However, mild toxicity was observed above 100 μg/ml for HepaRG cells, which could be due to the differences in the cells and their susceptibility to SFE. SFE protected cells against t-BHP-induced oxidative stress and increased cell viability in a dose-dependent manner up to 0.5 mg/ml. However, no further increase in cell viability was observed above this concentration. Our results are in line with Fernandes et al. who reported that SF hot water extract, up to concentrations of 40 μg/ml, had no adverse effect on the viability of human neutrophils after a 30 min treatment [12]. Our results are also supported by another study on proximal and distal convoluted tubule epithelial cell lines (LLC-PK1 and MDBK), in which the cell viability of both cell lines treated with concentrations between 6 mg/ml and 0.3 mg/ml for 48 h was more than 89% [32]. Results from Ngcobo et al. are similar to our results where they showed that although high concentrations of SF extracts (ethanol) can be toxic to normal T cells, SFW (water) fractions were relatively non-toxic. They found that 0.5 mg/ml SFW extract showed 81% live cells after 24 h [33]. In addition, safety studies in vervet monkeys and humans have suggested that SF extracts are not toxic [34, 35].

In contrast, some studies have shown cytostatic and cytotoxic effects of SF extracts in cervical carcinoma cells, Chinese hamster ovary cancer cells, Caski and Jurkat T Lymphoma cells, human breast adenocarcinoma (MCF-7), human non-tumorigenic epithelial mammary gland cells (MCF-12A), MDA-MB-468 cell line, human leukemia Jurkat cells, human promyelocyte HL60 cells, MDA-MB-231 breast cancer cells, DU-145 prostate cancer cells, and proximal and distal convoluted tubule epithelial cell lines (LLC-PK1 and MDBK) [32, 36–39].

These contrasting results could be due to a variety of factors such as differences in cell lines, preparation of extracts (tablets vs. dried plant parts), the extraction solvent, dosage concentrations and times, as well as varying components in plants grown in regions with different soil compositions and environmental factors (leading to synthesis and accumulation of secondary metabolites) [40].

Our results showed that SFE protected cells by scavenging ROS in a dose dependent manner in all three cell lines. These results are also in line with Fernandes et al., who reported that the SF hot water extract significantly decreased both the luminal and lucigenin enhanced chemiluminescence responses of neutrophils stimulated by FMLP in a dose related manner [12].

To further elucidate the mechanism of protection against t-BHP-induced oxidative stress, GSH and GSSG levels were measured. GSH, in its reduced form, is the most powerful intracellular antioxidant and the ratio of reduced to oxidized glutathione (GSH/GSSG) is representative of the antioxidative capacity of the cell. An increase in ROS, together with a decrease in GSH, sets off a cascade of further oxidative damage. SFE was able to prevent depletion of GSH in all the three cell lines. Our results are supported by a study done by Ngcobo [41] where the SF extracts decreased both cell viability and GSH levels in H9 cancerous cells while the same extracts significantly increased cell viability and GSH levels in normal T cells. The extracts caused a time-dependent decrease in GSH content in H9 cells with the SF water extract dilutions being more effective than the ethanol extracts. However, in normal T cells, the extracts negatively affected the levels of GSH at higher concentrations but enhanced the GSH content at lower concentrations. The SF water extract dilutions were also more effective in increasing the GSH content of normal T cells than the ethanol extracts. However, contrary to this, a significant decrease in GSH was reported in SF-treated MDBK cells and LLC-PK1 cells [32]. These contrary results could, again, be due to differences in cell lines, doses, incubation times, as well as extract preparation.

## Conclusion

Our results indicate that hot water is a better solvent for the extraction of the antioxidant ingredients of SF vegetative material. Flavonoids may be the key component responsible for the antioxidant potential of SF based on its superior radical scavenging ability. In addition, protection against t-BHP-induced oxidative stress in transformed as well as normal cell lines further demonstrates its antioxidant potential. *In vitro* assays indicate that this plant extract is a significant source of natural antioxidants, which might be helpful in preventing the progression of various oxidative stresses.

## References

[1] Hayes JD, McLellan LI (1999). Glutathione and glutathione-dependent enzymes represent a co-ordinately regulated defence against oxidative stress. Free Radic Res.

[2] Nakabeppu Y, Tsuchimoto D, Furuichi M, Sakumi K (2004). The defense mechanisms in mammalian cells against oxidative damage in nucleic acids and their involvement in the suppression of mutagenesis and cell death. Free Radic Res.

[3] Papas AM (1999). Diet and antioxidant status. Food Chem Toxicol.

[4] Morel I, Lescoat G, Cillard P, Cillard J (1994). Role of flavonoids and iron chelation in antioxidant action. Methods Enzymol.

[5] Rice-Evans C (2001). Flavonoid antioxidants. Curr Med Chem.

[6] Krinsky NI (1992). Mechanism of action of biological antioxidants. Proc Soc Exp Biol Med.

[7] van Wyk BE, Albrecht C (2008). A review of the taxonomy, ethnobotany, chemistry and pharmacology of Sutherlandia frutescens (Fabaceae). J Ethnopharmacol.

[8] Robak J, Gryglewski RJ (1988). Flavonoids are scavengers of superoxide anions. Biochem Pharmacol.

[9] Chen JW, Zhu ZQ, Hu TX, Zhu DY (2002). Structure-activity relationship of natural flavonoids in hydroxyl radical-scavenging effects. Acta Pharmacol Sin.

[10] Rice-Evans C, Packer L (1998). Flavonoids in health and disease.

[11] Fu X, Li XC, Wang YH, Avula B, Smillie TJ, Mabusela W, Syce J, Johnson Q, Folk W, Khan IA (2010). Flavonol glycosides from the south African medicinal plant Sutherlandia frutescens. Planta Med.

[12] Fernandes AC, Cromarty AD, Albrecht C, van Rensburg CE (2004). The antioxidant potential of Sutherlandia frutescens. J Ethnopharmacol.

[13] Katerere DR, Eloff JN (2005). Antibacterial and antioxidant activity of Sutherlandia frutescens (Fabaceae), a reputed anti-HIV/AIDS phytomedicine. Phytother Res.

[14] Koleva II, van Beek TA, Linssen JP, de Groot A, Evstatieva LN (2002). Screening of plant extracts for antioxidant activity: a comparative study on three testing methods. Phytochem Anal.

[15] Konaté K, Kiendrébéogo M, Ouattara MM, Souza A, Lamien-Meda A, Nongasida Y, Barro N, Millogo-Rasolodimby J, Nacoulma OG (2011). Antibacterial potential of aqueous acetone extracts from five medicinal plants used traditionally to treat infectious diseases in burkina faso. Curr Res J Biol Sci.

[16] Kalava V, Menon S (2012). In-vitro free radical scavenging activity of aqueous extract from the mycella of volvariella volvacea (bulliard ex fries) singer. Int J Current Pharmaceutical Res.

[17] Jayanthi P, Lalitha P (2011). Reducing Power of the solvent extracts of eichhornia crassipes (Mart.) solms. Int J Pharmacy Pharmaceutical Sci.

[18] Shyamala S, Vasantha K (2010). Free radical scavenging and antioxidant activity of leaves from Agathi (Sesbania grandiflora) (L.) Pers. Am Eurasian J Sci Res.

[19] Ozyurek M, Bektasoglu B, Guclu K, Gungor N, Apak R (2010). A novel hydrogen peroxide scavenging assay of phenolics and flavonoids using cupric reducing antioxidant capacity (CUPRAC) methodology. J Food Compos Anal.

[20] Pramod K, Devala RG, Lakshmayya B, Ramachandra SS (2011). Nephroprotective and nitric oxide scavenging activity of tubers of momordica tuberosa in rats. Avicenna J Med Biotechnol.

[21] Bajpai VK, Sharma A, Kang SC, Baek KH (2014). Antioxidant, lipid peroxidation inhibition and free radical scavenging efficacy of a diterpenoid compound sugiol isolated from Metasequoia glyptostroboides. Asian Pac J Trop Med.

[22] Kunchandy E, Rao MNA (1990). Oxygen radical scavenging activity of curcumin. Int J Pharm.

[23] Manda K, Adams C, Ercal N (2010). Biologically important thiols in aqueous extracts of spices and evaluation of their *in vitro* antioxidant properties. Food Chem.

[24] Bradford MM (1976). A rapid and sensitive method for the quantitation of microgram quantities of protein utilizing the principle of protein-dye binding. Anal Biochem.

[25] Wang B-J, Lien Y-H, Yu Z-R (2004). Supercritical fluid extractive fractionation – study of the antioxidant activities of propolis. Food Chem.

[26] Stohs SJ, Bagchi D (1995). Oxidative mechanisms in the toxicity of metal ions. Free Radic Biol Med.

[27] Marcocci L, Maguire JJ, Droy-Lefaix MT, Packer L (1994). The nitric oxide-scavenging properties of Ginkgo biloba extract EGb 761. Biochem Biophys Res Commun.

[28] Wink DA, Kasprzak KS, Maragos CM, Elespuru RK, Misra M, Dunams TM, Cebula TA, Koch WH, Andrews AW, Allen JS, Keefer LK (1991). DNA deaminating ability and genotoxicity of nitric oxide and its progenitors. Science.

[29] Halliwell B (1991). Reactive oxygen species in living systems: source, biochemistry, and role in human disease. Am J Med.

[30] Cook N, Samman S (1996). Flavonoids—Chemistry, metabolism, cardioprotective effects, and dietary sources. J Nutr Biochem.

[31] Benov L (2001). How superoxide radical damages the cell. Protoplasma.

[32] Phulukdaree A, Moodley D, Chuturgoon A (2010). The effects of Sutherlandia frutescens extracts in cultured renal proximal and distal tubule epithelial cells. S Afr J Sci.

[33] Ngcobo M, Gqaleni N, Chelule PK, Serumula M, Assounga A (2012). Effects of Sutherlandia frutescens extracts on normal T-lymphocytes *in vitro*. Afr J Tradit Complement Altern Med.

[34] Seier JV, Mdhluli M, Dhansay M, Loza J, Laubscher R (2002). A toxicity study of Sutherlandia leaf powder (Sutherlandia microphylla) consumption. Medical Research Council, National Research Foundation (NRF) of South Africa.

[35] Johnson Q, Syce J, Nell H, Rudeen K, Folk WR (2007). A randomized, double-blind, placebo-controlled trial of Lessertia frutescens in healthy adults. PLoS Clin trials.

[36] Stander A, Marais S, Stivaktas V, Vorster C, Albrecht C, Lottering ML, Joubert AM (2009). *In vitro* effects of Sutherlandia frutescens water extracts on cell numbers, morphology, cell cycle progression and cell death in a tumorigenic and a non-tumorigenic epithelial breast cell line. J Ethnopharmacol.

[37] Tai J, Cheung S, Chan E, Hasman D (2004). *In vitro* culture studies of Sutherlandia frutescens on human tumor cell lines. J Ethnopharmacol.

[38] Chinkwo KA (2005). Sutherlandia frutescens extracts can induce apoptosis in cultured carcinoma cells. J Ethnopharmacol.

[39] Vorster C, Stander A, Joubert A (2012). Differential signaling involved in Sutherlandia frutescens-induced cell death in MCF-7 and MCF-12A cells. J Ethnopharmacol.

[40] Wink M, Wiley InterScience (Online service) (2010). Functions And Biotechnology Of Plant Secondary Metabolites. Book Functions And Biotechnology Of Plant Secondary Metabolites.

[41] Nycobo M (2008). The biochemical effects of Sutherlandia Frutescens in cultured H9 cancerous T cells and normal human T lymphocytes. MMedSci Thesis, University of KwaZulu-Natal.

[CR42] The pre-publication history for this paper can be accessed here: http://www.biomedcentral.com/1472-6882/14/271/prepub

